# Management and treatment of relapsed or refractory Ph(−) B-precursor ALL: a web-based, double-blind survey of EU clinicians

**DOI:** 10.1186/s12885-015-1745-4

**Published:** 2015-10-24

**Authors:** Deborah Saltman, Arie Barlev, Divyagiri Seshagiri, Ioannis Katsoulis, Vincent Lin, Beth Barber

**Affiliations:** 1 PRMA Consulting, Fleet, UK; 2 School of Public Health, Imperial College, London, UK; 3 Amgen GHE, Thousand Oaks, USA

**Keywords:** Agents, R/R Ph(–) B-precursor ALL, Regimens, Salvage therapy, Treatment guidelines

## Abstract

**Background:**

The prognosis for adult patients with Ph(-) B-precursor acute lymphoblastic leukaemia (ALL) who are refractory to treatment or experience relapse (R/R), is poor; over 90 % of these patients die from the disease, typically within a few months. While there are some national guidelines published for the treatment of adult patients with ALL, and local working group recommendations do exist, there is very little detail and no preferred treatment regimens for adult patients with R/R Ph(-) B-precursor ALL. The aim of this study was to describe current real-world clinical practice in Europe for the management and treatment of adult R/R Ph(–) B-precursor ALL.

**Methods:**

A web-based, double-blind survey was conducted in November/December 2013 in France, Germany, Italy, Spain, and the UK. The survey was developed following consultation with specialist clinicians and a critical review of published literature. Eligible clinicians (15 per country) were board-certified in haemato-oncology or haematology; had at least 4 years of experience in their current role and had treated at least five patients with adult ALL in the 36 months before the survey, including at least one with R/R Ph(−) B-precursor ALL.

**Results:**

Clinicians across the five countries consulted 16 guidelines and local working group recommendations for the diagnosis and treatment of R/R Ph(–) B-precursor ALL. Thirty three regimens for salvage therapy were reported; the most frequently cited was augmented hyper-CVAD (15 %), with vincristine the most commonly used agent. Salvage therapy regimens involved a range of agents, and most respondents reported using at least one cytotoxic agent; across respondents 10 different cytotoxic agents were cited. All respondents reported that toxicity was common for the regimens they used to treat R/R Ph(–) B-precursor ALL.

**Conclusions:**

This study provides evidence of current management and treatment patterns of R/R Ph(–) B-precursor ALL in the real-world clinical practice in Europe. The approach to the treatment of R/R Ph(–) B-precursor ALL is heterogeneous, reflecting the lack of any clearly superior chemotherapeutic option, thus it appears that clinicians are trying a wide variety of therapies. These findings show a clear need for effective, tolerable treatments for R/R Ph(–) B-precursor ALL.

**Electronic supplementary material:**

The online version of this article (doi:10.1186/s12885-015-1745-4) contains supplementary material, which is available to authorized users.

## Background

Acute lymphoblastic leukemia (ALL) is a rare disease with an incidence of 1.2–1.4 per 100,000 population per year in Europe [1]. It is an aggressive malignancy, characterised by a sudden onset and rapid progression, and diagnosis usually requires urgent medical attention [2–4]. ALL encompasses several subtypes that are classified according to cell lineage (T- or B-cell), cell type (mature or precursor), and the presence or absence of the Philadelphia (Ph) chromosome translocation – Ph(+) or Ph(−).

The prognosis for adult patients with Ph(−) B-precursor ALL who are refractory to treatment or experience relapse is poor [5–9]. In a study incorporating all subtypes of ALL, ≥90 % of those with Ph(−) disease and those with the B-precursor immunophenotype died of the disease, with a 5 year overall survival of ≤10 % [10]. Median overall survival after a diagnosis of relapsed or refractory (R/R) disease is less than 6 months [9–12]. Unfortunately, progress in the development of treatments for Ph(−) B-precursor ALL has been slow, with scarce innovative treatments having been approved in the EU for adult patients in decades. Because of the lack of innovative treatment options, the only options for most patients are salvage therapy regimens and recourse to a wide range of chemotherapies used in combination, including corticosteroids, alkylating agents, anthracyclines and cytotoxic antibiotics, antimetabolites, vinca alkaloids, and asparaginase [5, 9]. The small percentage of patients who respond to salvage therapy, may have an option to receive allogeneic haematopoietic stem cell transplant (HSCT), which is currently the only potentially curative option for adult patients with R/R B-precursor ALL [13].

While there are some national and international guidelines published for treatment of adult patients with ALL (eg, NCCN 2014 [9]), there is very little detail and no preferred treatment regimens for adult patients with R/R Ph(−) B-precursor ALL. The guidelines list options for R/R Ph(−) B-precursor ALL, but do not recommend any one treatment over another. Although there are no Europe-wide clinical practice guidelines, recommendations have been proposed by the European Working Group for Adult Acute Lymphoblastic Leukemia (EWALL) [5]; but again, there is very little specific information about the treatment of adult R/R Ph(−) B-precursor ALL. In some countries, clinicians use treatment protocols developed by their respective national study groups for guidance, which may provide more specific information about the treatment of adult patients with R/R Ph(−) B-precursor ALL.

The literature and treatment guidelines acknowledge many options, however, there are no journal articles describing real-world treatment patterns. Therefore, the aim of this clinician survey was to describe current real-world clinical practice for the management – including diagnosis, response definition and HSCT eligibility – and treatment of R/R Ph(–) B-precursor ALL in five European countries (France, Germany, Italy, Spain, and the UK).

## Methods

A cross-sectional, web-based, double-blind survey of clinicians who specialise in the treatment of adults with ALL was conducted over an 8-week period in November/December 2013. The survey aimed to yield information on the diagnosis and treatment of patients with R/R Ph(–) B-precursor ALL in France, Germany, Italy, Spain, and the UK. Ethics approval was granted by the Human Research Ethics Committee of the University of Technology, Sydney and all participants provided informed consent before starting the survey. Eligible participants were compensated for their time (at the fair market value), provided this was allowed by local legislation and guidelines.

### Survey development

The survey was a structured questionnaire that was developed in three distinct and iterative steps [14].

#### Questionnaire development

The content of the questionnaire was informed by a critical review of clinical guidelines, treatment protocols for ALL, published papers on treatment practices in ALL, and systematic reviews of safety and efficacy studies [9, 15–33]. Haematologists and haemato-oncologists were then consulted to advise on the content. The questionnaire consisted mainly of multiple-choice questions with a few open-ended questions, and covered the following topics for R/R Ph(–) B-precursor ALL: demographics of responders; diagnosis; salvage therapy; evaluation of response; and use of HSCT. Please see abbreviated version of questionnaire as an additional file (Additional file 1).

#### Questionnaire validity

The questionnaire was pre-tested by haemato-oncologists from France, Germany and the UK to ensure the questions were relevant, comprehensible and unambiguous. A pilot questionnaire was then created based on the feedback received on the validity of the questionnaire and on input from a medical statistician.

#### Pilot study

The pilot study was conducted to confirm that the questionnaire was easily understood, and well targeted, and that it provided informative results. One participant was recruited per country, except for Germany where two participants were recruited, because Germany has the largest clinician population.

The pilot questionnaire was administered in the local language; the accuracy of translations was confirmed using back-translations. The web-based questionnaire was followed by a telephone interview in English to obtain feedback on the face validity, comprehensiveness, length, clarity, flow, ease of use, and design of the web interface of the questionnaire; the appropriateness of the clinical aspects of the questions and the accuracy of the translations were also assessed. Feedback from the pilot questionnaire was used to refine the questionnaire for the survey. Results from the pilot study were not included in the survey results and participants who were included in the pilot study were not included in the survey.

### Survey conduct

Eligible participants (15 per country) completed the web-based questionnaire at their own pace and in the local language. Participants were able to withdraw from the survey at any time and for any reason and were replaced with other eligible participants from the same country who met the eligibility criteria. Data from incomplete surveys were not used in the final analysis.

### Participant recruitment

Participants from France, Germany, Italy, Spain, and the UK were recruited from sampling lists. The lists were provided by a commercial agency with an established panel of practicing clinicians who treat adult ALL and have agreed to participate in such studies; the agency identified potential clinicians primarily through publications and attendance at conferences. Within each country, clinicians with the most experience of treating ALL were contacted first; contact and screening of potential clinicians continued until the planned number of eligible clinicians had completed the survey. An initial e-mail invitation was sent to clinicians explaining the objective of the study and a screener questionnaire then followed to determine clinicians’ eligibility to participate in the survey. Eligible participants were those who were board-certified in haemato-oncology or haematology; had at least 4 years’ experience in their current role (or similar role at another institution) and had treated at least five patients with adult ALL in the 36 months before the survey, including at least one patient with B-cell ALL, one with precursor B-cell ALL, one with Ph(-) B-precursor ALL and one with R/R Ph(–) B-precursor ALL.

The planned sample size of 15 participants per country was based on previous experience of working with this group of clinicians and the relatively small number of clinicians who treat patients with this disease. Although more than 100 oncologists and haemato-oncologists are registered with the agency for each country, the survey focused on a subgroup of patients with a rare disease, the screening criteria were restrictive, and the typical response rate for such surveys is 30 %; thus, 15 participants per country was expected to be achievable. Regional quotas were applied within each country based on the distribution of the general population.

### Statistical analysis

The survey was a descriptive study, and no formal statistical hypotheses were tested. Descriptive statistics were used to summarise findings and to enumerate the common regimens prescribed by clinicians who treat patients with R/R Ph(–) B-precursor ALL. Percentage and number of respondents were used to describe categorical variables. Mean and median were used to describe continuous variables. For some questions respondents could select multiple criteria resulting in statistics that were not mutually exclusive. De-identified datasets from the survey were used to analyse the results for each country separately; when appropriate, the results from the five countries were then aggregated. The analyses were performed by a statistician using SAS statistical package version 9.3 (Cary, NC), according to a pre-specified statistical analysis plan.

## Results

### Demographics of responders

A total of 187 clinicians were contacted (France, *n* = 38; Germany, *n* = 28; Italy, *n* = 50; Spain, *n* = 32; UK, *n* = 39); of these, 23 were not eligible because they did not meet the screening criteria, either because they did not treat haematological malignancies frequently; had not treated enough patients in the last 36 months; or had <4 years’ experience in treating ALL (France, *n* = 3; Germany, *n* = 1; Italy, *n* = 8; Spain, *n* = 2; UK, *n* = 9). Of those who were eligible (*n* = 164), 75 clinicians across the five countries completed the survey (overall completion rate for eligible clinicians, 46 %; completion rates per country: France, 43 %; Germany, 56 %; Italy, 36 %; Spain, 50 %; UK, 50 %). Most respondents worked at university hospitals (64 %, *n* = 48) or at cancer hospitals/specialist oncology centres/specialist haematology centres (23 %, *n* = 17). The demographics of those clinicians who were eligible and who completed the survey (*n* = 15, per country) are shown in Table 1.

**Table 1:** Demographics of respondents in each country (*N* = 15 per country)

	France	Germany	Italy	Spain	UK	Total
	*N* = 15	*N* = 15	*N* = 15	*N* = 15	*N* = 15	*N* = 75
Speciality, *n* (%)						
Oncologist	5 (33)	1 (7)	3 (20)	0 (0)	0 (0)	9 (12)
Haematologist	5 (33)	2 (13)	7 (47)	8 (53)	8 (53)	30 (40)
Haemato-oncologist	5 (33)	12 (80)	5 (33)	7 (47)	7 (47)	36 (48)
Location, *n* (%)						
University hospital	7 (47)	10 (67)	7 (47)	13 (86)	11 (73)	48 (64)
Cancer hospital/specialist centre	6 (40)	4 (27)	5 (33)	0 (0)	2 (13)	17 (23)
Community/urban/general hospital	2 (13)	1 (7)	3 (20)	2 (13)	2 (13)	10 (13)
Experience, years						
Years treating adults with ALL, median (range)	15 (7–29)	12 (8–18)	10 (6–30)	16 (9–34)	12 (17–20)	N/A
Caseload^a^ of ALL subtypes from 12 months before the survey, *n*						
ALL, median (range)	30 (3–200)	20 (6–100)	30 (5–100)	20 (3–230)	24 (4–200)	N/A
R/R Ph(–) B-precursor ALL, median (range)	8 (1–75)	3 (1–21)	5 (1–50)	4 (0–55)	4 (1–50)	N/A

^a^Number of patients

*ALL* acute lymphoblastic leukaemia, *N/A* not available, *Ph(–)* Philadelphia negative, *R/R* relapsed or refractory

### Diagnosis of R/R Ph(–) B-precursor ALL

The clinical criteria used to define relapsed or refractory disease were similar across the countries. For relapsed disease the most frequently reported clinical criteria were analysis of lymphoblasts in bone marrow (BM) aspirate and biopsy (91 %, *n* = 68) and minimal residual disease (MRD) by either PCR or flow cytometry (FCM) (65 %, *n* = 49). For refractory disease the most frequently reported clinical criteria were also analysis of lymphoblasts in BM aspirate and biopsy (89 %, *n* = 67) and MRD by either PCR or FCM (69 %, *n* = 52). Most clinicians (73 %, *n* = 55) consulted treatment guidelines and/or local working group recommendations for advice on making a diagnosis of R/R Ph(−) B-precursor ALL, with 10 guidelines and local working group recommendations cited (Table 2).

**Table 2:** Guidelines/local working group recommendations consulted for diagnosis of relapsed or refractory Ph(–) B-precursor ALL

Guideline or local working group recommendation	Number of respondents consulting for diagnosis, *n* (%)^a^					
	France	Germany	Italy	Spain	UK	Total
	*N* = 15	*N* = 15	*N* = 15	*N* = 15	*N* = 15	*N* = 75
ASH/ASCO, US	6 (40)	2 (13)	10 (67)	5 (33)	3 (20)	26 (35)
EWALL, Europe	6 (40)	1 (7)	3 (20)	2 (13)	4 (27)	16 (21)
NCCN, US	3 (20)		7 (47)	4 (27)		14 (19)
GMALL, Germany	1 (7)	8 (53)	1 (7)	1 (7)		11 (15)
GIMEMA, Italy			10 (67)			10 (13)
PETHEMA, Spain				9 (60)		9 (12)
Others^b^	3 (20)				1 (7)	4 (5)

^a^Respondents could select multiple guidelines

^b^The following guidelines and/or local working group recommendations were consulted by one respondent for diagnosis: HAS, France; Onco LR, France; GELA, France; BCSH, UK

ALL, acute lymphoblastic leukaemia; ASCO, American Society of Clinical Oncology; ASH, American Society of Hematology; BCSH, the British Committee for Standards in Haematology; EWALL, European Working group for Adult Lymphoblastic Leukemia; GELA, Groupe d’Etude des Lymphomes de l’Adulte (Study Group of the Adult Lymphoma); GIMEMA, Gruppo Italiano Malattie EMatologiche dell’Adultodell’Adulto (Italian Group for Haematological Diseases in Adults); GMALL, German Multicenter Study Group for Adult ALL; HAS, Haute Autorité de Santé (High Authority for Health); NCCN, the National Comprehensive Cancer Network; Onco LR, le réseau régional de Cancérologie en Languedoc Roussillon (regional network of Oncology in Languedoc Roussillon); PETHEMA, Program for Study and Treatment of Malignant Haemopathies, Spanish Society of Haematology

### Treatment patterns of R/R Ph(–) B-precursor ALL

#### Treatment guidelines

Across the five countries, no central guideline for the treatment of R/R Ph(−) B-precursor ALL is consulted. Most clinicians consult local working group recommendations (i.e. the German Multicenter Study Group for Adult ALL [GMALL]; the Gruppo Italiano Malattie EMatologiche dell’Adultodell’Adulto [GIMEMA]; the Program for Study and Treatment of Malignant Haemopathies, Spanish Society of Haematology [PETHEMA], and the guidelines of the Medical research Council ALL Trials XII [UKALL XII]) (Table 3). However, more than half of the clinicians refer to American Society of Hematology/American Society of Clinical Oncology [ASH/ASCO] guidelines, in addition to their local working group recommendations. EWALL and National Comprehensive Cancer Network (NCCN) guidelines were also frequently consulted. In total 16 guidelines and/or local working group recommendations were cited (Table 3).

**Table 3:** Guidelines/local working group recommendations consulted for treatment of relapsed or refractory Ph(–) B-precursor ALL

Guideline or local working group recommendation	Number of respondents consulting for treatment n (%)^a^					
	France	Germany	Italy	Spain	UK	Total
	*N* = 15	*N* = 15	*N* = 15	*N* = 15	*N* = 15	*N* = 75
ASH/ASCO, US	9 (60)	3 (20)	7 (47)	6 (40)	4 (27)	29 (39)
EWALL, Europe	7 (47)	1 (7)	2 (13)	2 (13)	7 (47)	19 (25)
NCCN, US	6 (40)	1 (7)	5 (33)	5 (33)		17 (23)
GMALL, Germany	1 (7)	13 (60)	1 (7)	1 (7)		16 (21)
GIMEMA, Italy			12 (80)	1 (7)		13 (17)
PETHEMA, Spain				11 (73)	1 (7)	12 (16)
UKALL XII, UK			1 (7)		11 (73)	12 (16)
HAS, France	2 (13)					2 (3)
Others^b^	4 (27)				3 (20)	7 (9)

^a^Respondents could select multiple guidelines

^b^The following guidelines and/or local working group recommendations were consulted by one respondent for treatment: COOPRALL, France; Onco LR, France; GELA, France; GRAALL, France; BCSH, UK; The Beatson hospital, UK^c^; UKALL 2011 for young adults, UK; non-specified protocol

^c^The Beatson hospital is a cancer centre located in the West of Scotland

ALL, acute lymphoblastic leukaemia; ASCO, American Society of Clinical Oncology; ASH, American Society of Hematology; BCSH, the British Committee for Standards in Haematology; COOPRALL, Protocole Coopérateur de Traitement des Rechutes de Leucémies Aiguës Lymphoblastiques de L’enfant (treatment protocol for relapse of ALL in children); EWALL, European Working group for Adult Lymphoblastic Leukemia; GELA, Groupe d’Etude des Lymphomes de l’Adulte (Study Group of the Adult Lymphoma); GET-LALA, Groupe d’Etude et de Traitement des Leucémie Aiguës Lymphoblastique de l’Adulte (Group for the Study and Treatment of Adult ALL, France); GIMEMA, Gruppo Italiano Malattie EMatologiche dell’Adultodell’Adulto (Italian Group for Haematological Diseases in Adults); GMALL, German Multicenter Study Group for Adult ALL; GOELAMS, Groupe Ouest Est des Leucémies Aiguës et Maladies du Sang (East West Group of Acute Leukemia and Blood Diseases, France); GRAALL, Cooperative group combining the French GET-LALA, the GOELAMS, and the SAKK; HAS, Haute Autorité de Santé (High Authority for Health); NCCN, the National Comprehensive Cancer Network; Onco LR, le réseau régional de Cancérologie en Languedoc Roussillon (regional network of Oncology in Languedoc Roussillon); PETHEMA, Program for Study and Treatment of Malignant Haemopathies, Spanish Society of Haematology; SAKK, Schweizer Arbeitsgemeinschaft für klinische Krebsforschung, Leukämie-Arbeitsgruppe (Swiss Group for Clinical Cancer Research, Leukemia Working Group); UKALL 2011 for young adults, United Kingdom Trial for children and young adults with Acute lymphoblastic Leukaemia and Lymphoma 2011; UKALL XII, Medical Research Council Acute Lymphoblastic Leukemia Trial XII

#### Salvage therapy

Across the five countries, a range of salvage therapy regimens were reported by clinicians for the treatment of R/R Ph(−) B-precursor ALL; of 33 reported regimens, augmented hyper-CVAD (hyperfractionated-cyclophosphamide, vincristine, doxorubicin, dexamethasone) was the most frequently reported (15 %, *n* = 11), followed by FLAG-IDA (fludarabine, high-dose cytarabine-idarubicin) (12 %, *n* = 9) and GMALL 07/03 (9 %, *n* = 7) (Table 4). The majority of clinicians reported using published regimens of salvage therapy (81 %, *n* = 61); a smaller number of respondents reported using modified versions of published regimens (16 %, *n* = 12). Few respondents (3 %, *n* = 2) used regimens from ongoing clinical trials. The reported regimens consist of a range of agents. Cyclophosphamide, vincristine, prednisone and L-asparaginase were frequent components of the published regimens. Of the agents reported by clinicians across all regimens (published and modified), vincristine was the most commonly reported (61 %, *n* = 46) followed by L-asparaginase (49 %, *n* = 37). The use of at least one cytotoxic agent was reported by most respondents, particularly anthracyclines (88 %, *n* = 64); other agents included immunosuppressor/anti-inflammatory agents (i.e. dexamethasone and predniso(lo)ne; 68 %, *n* = 50), agents for the reduction of side effects (i.e. granulocyte-colony stimulating factor and folinic acid; 70 %, *n* = 51), additional central nervous system prophylaxis (66 %, *n* = 48), and the immunotherapy agent inotuzumab ozogamicin (1 %, *n* = 1). Most clinicians (70 %, *n* = 51) reported using one course of salvage therapy, although some (19 %, *n* = 14) also used a preparatory course and/or a second course (23 %, *n* = 17). When reporting the agents used for salvage therapy, two respondents were excluded as their description of the regimen used did not match the agents they had reported using (France, *n* = 1; UK, *n* = 1).

**Table 4:** Regimens used for salvage therapy, per country

Regimen	Number of respondents, n^a,b^					
	France	Germany	Italy	Spain	UK	Total, n (%)
	*N* = 15	*N* = 15	*N* = 15	*N* = 15	*N* = 15	*N* = 75
Published regimens						
Augmented hyper-CVAD	3	1	2	5		11 (15)
CALGB 8811/Larson	1	1	1			3 (4)
EORTC ALL-3 (induction phase)	1		2			3 (4)
FLAG-AMSA		1			1	2 (3)
FLAG-IDA		1		2	6	9 (12)
GMALL 07/03		6	1			7 (9)
GRAALL 02/2005 (salvage phase)	2	1				3 (4)
GRAALL 2003 (salvage phase)	1	2				3 (4)
Hyper-CVAD		1	1	1	1	4 (5)
MRC UK ALL XII/ECOG 2993			1		3	4 (5)
PETHEMA ALL-96				2		2 (3)
Modified published regimens						
FLAG-IDA				1	2	3 (4)

^a^Respondents could select multiple guidelines regimens

^b^Published regimens reported by one respondent were: EORTC ALL3 (salvage); GIMEMA 0288 (induction phase); GIMEMA 0288 (salvage); GIMEMA-LAL0904 (induction phase); GIMEMA-LAL0904 (salvage); GRAALL 02/2005 (induction phase); LALA-94; nelarabine/cyclophosphamide; PALG 5–2007 (Induction II); VANDEVOL; modified published regimens reported by one respondent were: aBFM; augmented hyper-CVAD; CALGB 8811/Larson; GRAALL 02/2005 (induction phase); LALA-94; MRC UK ALL XII/ECOG 2993; PETHEMA ALL-96; RWGALS – NP1 (Induction I); VANDEVOL; ongoing clinical trials reported by one respondent were: NCT01564784 and UKALL 2011 trial

aBFM, augmented Berlin-Frankfurt-Muenster; ALL, acute lymphoblastic leukaemia; CALGB, Cancer and Leukemia Group B; CVAD, cyclophosphamide, vincristine, doxorubicin (Adriamycin), dexamethasone; ECOG, Eastern Cooperative Oncology Group; EORTC, European Organization for Research and Treatment of Cancer*;* FLAG-AMSA, fludarabine, high-dose cytarabine, granulocyte colony-stimulating factor-amsacrine; FLAG-IDA, fludarabine, high-dose cytarabine, granulocyte colony-stimulating factor-idarubicin; GIMEMA, Gruppo Italiano Malattie EMatologiche dell’Adulto (Italian Group for Haematological Diseases in Adults); GMALL, German Multicenter Study Group for Adult ALL; GRAALL, Group for Research in Adult Acute Lymphoblastic Leukemia; LALA, Leucémie Aiguës Lymphoblastique de l’Adulte (Acute Lymphoblastic Leukemia in Adults); MRC UKALL XII, Medical Research Council Acute Lymphoblastic Leukemia Trial XII; PALG, Polish Adult Leukemia Group; PETHEMA, Programa para el Estudio de la Terapéutica en Hemopatía Maligna (Program for Study and Treatment of Malignant Hemopathies); RWGALS-NP1, Romanian Working Group for Acute Leukemia Study National Protocol 1; VANDEVOL, etoposide, clofarabine, asparaginase, mitoxantrone, and dexamethasone

#### Adverse events

All respondents reported that treatment- or disease-related toxicities were common. Cytopenia, infection and mucositis were reported to be the most frequent adverse events (AEs) by most respondents (91 %, 85 %, 76 % of clinians reported the AE as common, respectively). Other AEs reported to be quite frequent were pyrexia, fatigue, bleeding, neuropathy, cardiac toxicity and hepatopathy. Cyclophosphamide and high-dose cytarabine were among the individual agents most commonly reported to cause AEs (50 % and 36 % of all reported agents, respectively).

#### Assessment of response to salvage therapy

Assessment of response after completion of therapy was typically done after a median of 30-days (range: 2–180-days). The most commonly reported tests to evaluate response were complete blood count (85 %, *n* = 64), BM aspirate followed by FCM immunophenotyping (75 %, *n* = 56) and physical examination (72 %, *n* = 54). Other tests used by more than half of respondents were peripheral blood differential count (69 %, *n* = 52), BM aspirate/biopsy followed by cell morphology analysis (61 %, *n* = 46), peripheral blood smear (60 %, *n* = 45) and MRD by PCR (59 %, *n* = 44).

#### Complete remission

The most frequently chosen criterion to define CR was the level of lymphoblasts in BM (87 %, *n* = 65). Absence of circulating lymphoblasts (77 %, *n* = 58), absence of extramedullary disease (75 %, *n* = 56), and MRD using either PCR or FCM (69 %, *n* = 52) were also reported by more than 50 respondents. Most respondents (69 %, *n* = 52) considered CR to have been achieved if the relevant criteria were met at a time point after completion of treatment, regardless of how long the response was maintained, whereas some respondents (11 %, *n* = 8) required the response to be maintained for a specific time from completion of treatment. Other respondents required the response to be maintained from completion of treatment throughout further therapy (20 %, *n* = 15).

#### Eligibility for HSCT after salvage therapy

Most respondents (76 %, *n* = 57) reported that HSCT could be performed at their centre. Over the 36 months before the clinician survey, almost half of the respondents’ patients were deemed eligible for HSCT (48 %), and 42 % received HSCT.

The most frequently chosen criterion to determine eligibility for HSCT was the level of lymphoblasts in BM (81 %, *n* = 61). Most respondents (63 %, *n* = 47) considered a patient to be eligible for HSCT if the relevant criteria were met at a time point after completion of treatment, regardless of how long the response was maintained, whereas some respondents (12 %, *n* = 9) required the response to be maintained for a specific time from completion of treatment (range: 21 days to 12 months). Other respondents required the response to be maintained from completion of treatment throughout further therapy (25 %, *n* = 19).

## Discussion

To our knowledge, this is the first study to explore the real-world clinical practice for adult R/R Ph(–) B-precursor ALL in Europe. The results of this survey have shown that the approach to the management and treatment of R/R Ph(−) B-precursor ALL is heterogeneous. Across the five countries, 16 guidelines and local working group recommendations were consulted for the diagnosis and treatment of R/R Ph(–) B-precursor ALL. Clinicians reported the use of 33 regimens for salvage therapy with augmented hyper-CVAD the most frequently reported (15 %). Salvage therapy consisted of 10 different cytotoxic agents, with the use of at least one cytotoxic agent reported by most respondents.

The existence of so many regimens is likely to reflect the lack of a clearly superior chemotherapeutic option for the treatment of R/R Ph(–) B-precursor ALL. With no superior option available, it appears that clinicians who treat these patients are trying a wide variety of therapies to treat the disease, with it likely that treatment decisions may be impacted by access to certain drugs and patient-related factors (i.e. age and comorbidities), in addition to clinical efficacy. Of interest, few clinicians treat their patients within clinical trials which may reflect the scarcity of new agents and potential difficulty to access clinical trials. Although only 10 different cytotoxic agents were reported, the wide variety of regimens reflected different combinations, dosings, and dosing intervals of these agents, which further reflect the lack of a treatment consensus for treatment of R/R Ph(–) B-precursor ALL.

The reported timing of the assessment of response to salvage therapy also varied widely; although the median is 30 days, clinicians reported up to 180 days. It is postulated that these clinicians might be looking for a durable response to salvage therapy, thus the extended period prior to assessment. Similiarly, it is likely that clinicians were also looking for a durable response when considering their patient’s eligibility for HSCT, hence the wide range reported for maintenance of response from completion of treatment (range: 21 days to 12 months).

In addition to being the first study to explore the real-world clinical practice for adult R/R Ph(−) B-precursor ALL in Europe, there are several strengths in this study. First is the robust method of survey development. To develop the survey, a thorough review of guidelines and of the literature was conducted, as well as obtaining clinician input. We then conducted pre-testing and a pilot survey to ensure the validity of the survey. Second, we pre-specified the number of respondents for each country, to make sure the data we collected were representative and accurate. Third, we set up stringent inclusion criteria for the respondents to ensure the responses were from those who have extensive experience treating R/R Ph(−) B-precursor ALL.

There were some potential limitations to the study. The sampling process aimed to recruit clinicians with the most experience, and most were from specialist centres, such as university hospitals and cancer hospitals. However, not all eligible clinicians accepted the invitation to participate in the survey. For this reason, there is a potential for bias in the responses. Regarding sample size, although the pre-specified number of responders may be considered small (*n* = 15), given the rarity of the disease and the stringent inclusion criteria requiring clinicians to have treated a certain amount of ALL patients, the sample size is considered adequate.

## Conclusion

This study provides evidence of current management and treatment patterns of R/R Ph(–) B-precursor ALL in the real-world clinical practice. There is a lack of consensus and treatment for adults with this disease varies widely across Europe. The existence of so many regimens reflects the lack of a clearly superior chemotherapeutic option for the treatment of R/R Ph(–) B-precursor ALL; thus it appears that clinicians are trying a wide variety of therapies. These finding show a clear need for effective, tolerable treatments for R/R Ph(–) B-precursor ALL.

## Additional file

Additional file 1: **Abbreviated version of questionnaire.** (DOCX 33 kb)
