# Supplementary material for: Management and treatment of relapsed or refractory Ph(−) B-precursor ALL: a web-based, double-blind survey of EU clinicians
Source: BMC Cancer. 2015 Oct 24;15:771. doi: 10.1186/s12885-015-1745-4 (PMC4619337; doi:10.1186/s12885-015-1745-4)
Supplement: Additional file 1: — Abbreviated version of questionnaire. (DOCX 33 kb) [file 12885_2015_1745_MOESM1_ESM.docx]

**Additional file**

**Management and treatment of relapsed or refractory Ph(-) B-precursor ALL: a web-based, double-blind survey of EU clinicians**

**Treatment practice questions**

Introduction

1. What type(s) of specialists treat adult patients with refractory or relapsed stages of precursor B-cell acute lymphoblastic leukemia (ALL) in your country?
2. In the last 12 months, how many adult patients (≥18 years of age) with Ph~~–~~ precursor B-cell ALL and refractory or relapsed Ph~~–~~ precursor B-cell ALL did you or your team see?
3. In the last 12 months, how many of your adult patients with refractory or relapsed Ph– precursor B-cell ALL were treated in a clinical trial? Please provide the names and/or numbers of the trials.
4. In your practice, when do you define adult Ph– precursor B-cell ALL as refractory after induction therapy at first diagnosis? (in terms of number of regimens of induction completed and tests performed, and timing after treatment ends)
5. In your practice, when do you define adult Ph– precursor B-cell ALL has early relapse after induction therapy at first diagnosis? (in terms of failure of treatment at a specified time after treatment ends)
6. In your practice, when do you define adult Ph– precursor B-cell ALL has late relapse after induction therapy at first diagnosis? (in terms of failure of treatment at a specified time after treatment ends)
7. In your practice, what clinical criteria are necessary for you to define that an adult Ph–precursor B-cell ALL is refractory after induction therapy at first diagnosis?
8. In your practice, what clinical criteria are necessary for you to define that an adult Ph–precursor B-cell ALL has relapsed?
9. Do you follow any written guidelines to define refractory or relapsed adult Ph–precursor B-cell ALL?
10. What written guidelines do you follow to diagnose refractory or relapsed adult Ph–precursor B-cell ALL?
11. Please select all the types of tests and procedures that you typically use to inform your diagnosis of adult Ph– precursor B-cell ALL.

Treatment

1. In your experience, at what age would a young patient with refractory or relapsed Ph−precursor B-cell ALL stop being treated by a pediatrician? Please specify number in years.
2. Overall, what guidelines do you follow when treating adult patients with refractory or relapsed Ph− precursor B-cell ALL?
3. Do you monitor treatment response during treatment with induction regimens of salvage therapy for refractory or relapsed adult Ph– precursor B-cell ALL? Please provide details.
4. In your opinion, what are the clinical objectives of treatment for adult patients with refractory or relapsed Ph– precursor B-cell ALL?
5. In your practice, what induction regimen of salvage therapy would you normally administer an adult patient with refractory or relapsed Ph– precursor B-cell ALL? (Please describe treatment, including scheduling and duration)
   1. In the last 36 months, how many of your adult patients with refractory or relapsed Ph– precursor B-cell ALL were treated with this regimen?
6. What are the factors that you normally consider to choose the first induction regimen of salvage therapy for a patient with refractory or relapsed Ph– precursor B-cell ALL? Please choose how important each factor is to your practice.

*Second line treatment*

1. When an adult patient with refractory or relapsed Ph– precursor B-cell ALL who received salvage therapy with the above regimen does not achieve partial remission or complete remission, what is the next option that you normally recommend your patient? (Please describe treatment, including scheduling and duration or palliative care).
   1. In the last 36 months, how many of your adult patients with refractory or relapsed Ph– precursor B-cell ALL were treated with this regimen?
2. What are the factors that you normally consider to choose further salvage therapy for a patient with refractory or relapsed Ph– precursor B-cell ALL? Please choose how important each factor is to your practice.

*Third line treatment*

1. When an adult patient with refractory or relapsed Ph– precursor B-cell ALL who received salvage therapy with the above regimen does not achieve partial remission or complete remission, what is the next option that you normally recommend your patient? (Please describe treatment, including scheduling and duration or palliative care)
   1. In the last 36 months, how many of your adult patients with refractory or relapsed Ph– precursor B-cell ALL were treated with this regimen?

Consolidation regimen

1. What are the factors that you normally consider to choose a consolidation therapy regimen for a patient with refractory or relapsed Ph– precursor B-cell ALL? Please choose how important each factor is to your practice.
2. In your practice, what consolidation regimen do you most commonly use for adult patients with refractory or relapsed Ph− precursor B-cell ALL after a successful salvage therapy induction?

Maintenance regimen

1. In your practice, what maintenance regimen do you most commonly use for adult patients with refractory or relapsed Ph− precursor B-cell ALL? (Please describe treatment, including scheduling and duration)
   1. In the last 36 months, how many of your adult patients with refractory or relapsed Ph– precursor B-cell ALL were treated with maintenance treatment with the above regimen?

Stem cell transplantation (SCT)

1. Is stem cell transplantation (SCT) available at your center?
2. After providing salvage therapy to adult patients with refractory or relapsed Ph−precursor B-cell ALL, when do you normally treat these patients with SCT? (e.g. in terms of age threshold)
3. Please indicate the number of patients who were eligible for/recommended for/received any SCT after salvage therapy in your adult patients with refractory or relapsed Ph– precursor B-cell ALL from the last 36 months.

Adverse events

1. In your experience, what are the six most common adverse events observed in adult patients with refractory or relapsed Ph− precursor B-cell ALL who receive first induction for salvage therapy? Please describe according to regimens used, and specify if disease or treatment related.
